# Recurrent Benign Contrast-Induced Sialadenitis: A Case Report

**DOI:** 10.7759/cureus.107989

**Published:** 2026-04-29

**Authors:** Yuriko Iizuka, Eri Kamimura, Ichirou Kanetsuki, Masaya Hidaka, Yoshinori Morita, Tadahide Noguchi, Takeo Sato

**Affiliations:** 1 Department of Internal Medicine, Kagoshima Prefecture Oshima Hospital, Amami, JPN; 2 Department of Radiology, Kagoshima Prefecture Oshima Hospital, Amami, JPN; 3 Department of Dentistry, Oral and Maxillofacial Surgery, Jichi Medical University, Tochigi, JPN; 4 Department of Medicine, Division of Rheumatology and Clinical Immunology, Jichi Medical University, Tochigi, JPN

**Keywords:** contrast induced sialadenitis, contrast-induced sialadenitis, corticosteroid, iodide mumps, iodinated contrast media, ultrasound (u/s)

## Abstract

Contrast-induced sialadenitis (CIS) is a rare, benign condition characterized by painless salivary gland swelling after iodinated contrast administration. A 60-year-old man developed bilateral neck swelling 14 hours post-contrast-enhanced computed tomography (CT). CIS was diagnosed based on characteristic ultrasonographic findings, namely, salivary gland enlargement with increased blood flow, which resolved spontaneously within three days. Despite corticosteroid premedication before a subsequent contrast-enhanced CT four months later, similar symptoms recurred. The effectiveness of corticosteroids in preventing CIS remains uncertain. This case highlights the potential for CIS recurrence and the need for further investigation to establish optimal management and preventive strategies.

## Introduction

Contrast-induced sialadenitis (CIS) is a rare condition characterized by rapid, painless swelling of the neck occurring several hours after exposure to contrast media. Fewer than 100 clinically overt cases have been reported worldwide, with only two cases previously reported in Japan [1,2]. Although the reported incidence of clinically apparent CIS is generally estimated to be 1-2% [3], Lee et al. [4] reported an incidence of 4.2% in a cohort of 780 patients who underwent endovascular treatment for cerebrovascular disease. This discrepancy suggests that mild or subclinical cases may be underrecognized or underreported. Therefore, CIS may be more common than clinicians currently recognize.

The detailed pathophysiology of CIS remains unclear. However, iodinated contrast media are primarily excreted by the kidneys; a small proportion may be excreted through the salivary glands and accumulate within them, leading to localized inflammation and edema owing to increased vascular permeability [5,6]. Neck ultrasonography is a useful diagnostic tool, as CIS often presents with characteristic imaging findings, including bilateral submandibular gland enlargement with internal hypoechoic tubular structures and increased central blood flow [6]. Anti-inflammatory agents, including nonsteroidal anti-inflammatory drugs and corticosteroids, have been used to treat CIS, but most cases have resolved spontaneously without intervention [7].

CIS may recur upon re-exposure to contrast media. However, the efficacy of corticosteroid premedication for preventing CIS remains controversial. Here, we report a case of recurrent CIS despite corticosteroid premedication and the use of a different iodinated contrast agent, providing further insight into the recurrence and the limitations of preventive strategies for this rare condition.

## Case presentation

A 60-year-old man was referred to our hospital for further evaluation of a gallbladder polyp detected during a routine abdominal ultrasound examination. The patient had a history of untreated hypertension and mild renal impairment but no other significant medical conditions or known allergies. After receiving a 500 mL saline infusion because of mild renal impairment, he underwent contrast-enhanced computed tomography (CT) using 100 mL of iopamidol 370. No adverse reactions were observed immediately after the examination, and the patient returned home. Fourteen hours after contrast administration, he developed bilateral neck swelling and presented to the emergency department at our hospital.

**Figure 1:**
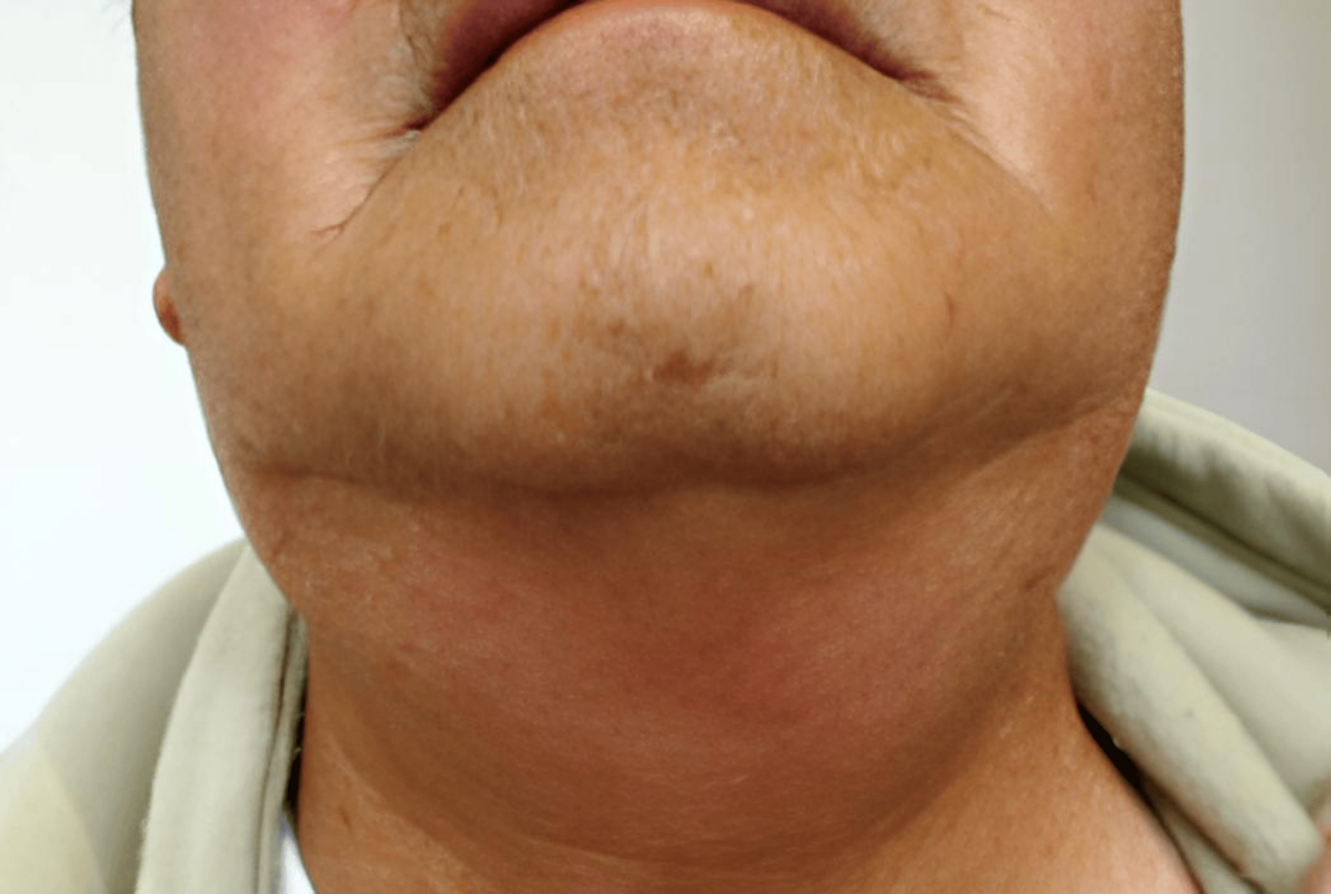
Bilateral swelling of the salivary glands after iopamidol infusion (hospital day 1 (HOD1))

On arrival, his general appearance was good; he was afebrile, hemodynamically stable, and showed no hypotension. Physical examination revealed marked bilateral neck swelling with a soft, elastic consistency and no tenderness (Figure 1).

No lip edema, rash, wheezing, or respiratory distress was observed. Laboratory test results are shown in Table 1. There were no findings indicating significant leukocytosis or systemic inflammation. Mild renal impairment was noted.

**Table 1:** Laboratory test results

Parameters	Measured value	Reference range
White blood cell count (WBC)	11,320	(3600-9000)/μL
Eosinophil fraction	4.40	(0-5)%
C-reactive protein level	0.22	(≦0.3 ) mg/dL
Creatinine	1.13	(≦1.00) mg/dL
Blood urea nitrogen	17.70	(7-24) mg/dL
Estimated glomerular filtration rate	52.40	(≧60) mL/min/1.73 m²
Amylase	237	(44-132) U/L
P-type amylase	47	(18-53) U/L
S-type amylase	152	(6-84) U/L
T-type amylase	199	(44-132) U/L
Total serum IgE	723	(≦174) U/L
IgG subclass 4	62.40	(11-121) U/L

**Figure 2:**
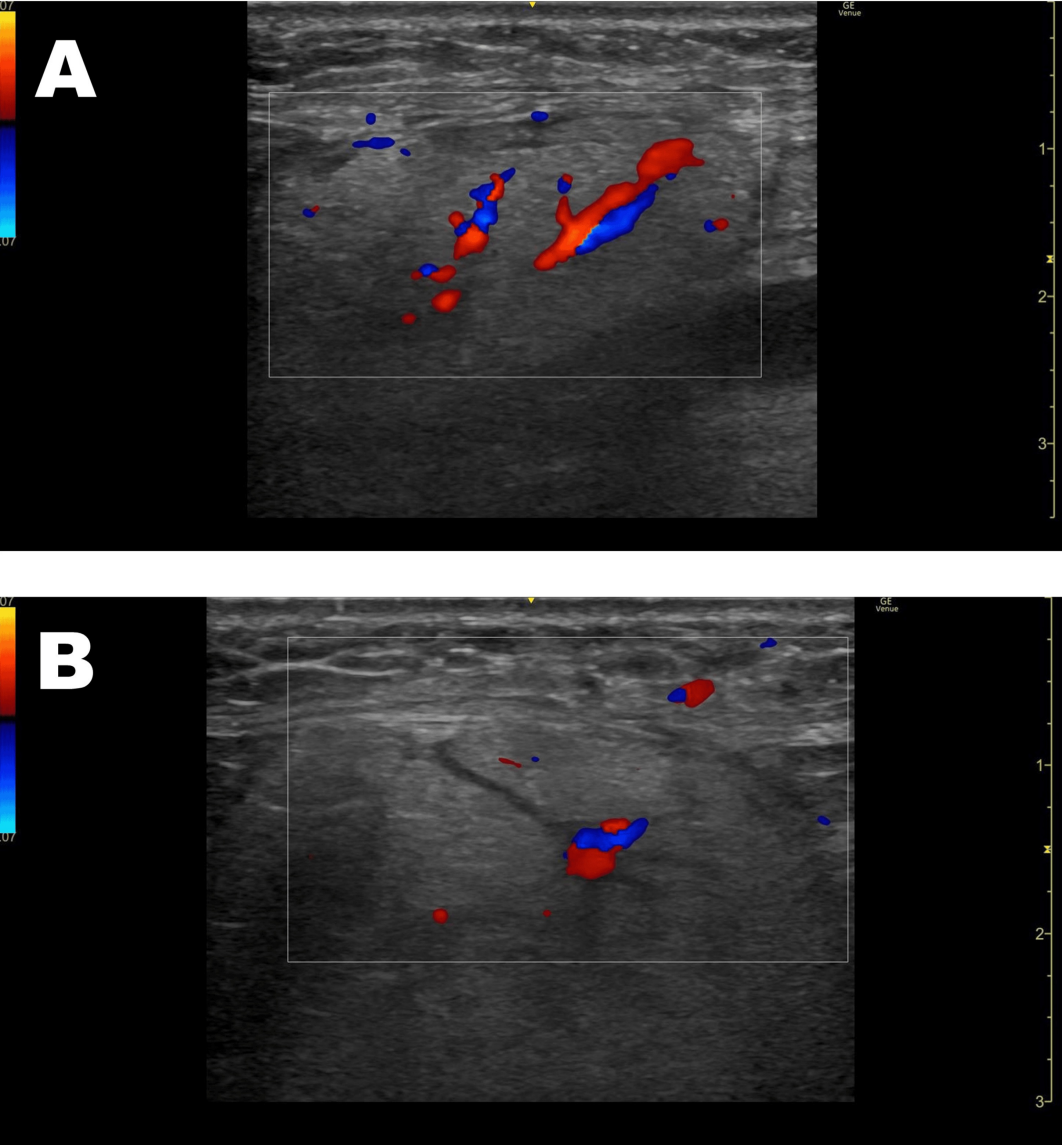
Neck color Doppler ultrasound findings (HOD1). (A) Right submandibular gland. (B) Left submandibular gland Bilateral submandibular gland enlargement with tubular structures suggestive of salivary duct involvement is observed. Color Doppler imaging shows increased blood flow in the central region of the submandibular glands. HOD1: hospital day 1

Neck ultrasonography showed bilateral submandibular gland enlargement with tubular structures suggestive of salivary duct involvement. Color Doppler imaging showed increased blood flow in the central region of the submandibular glands (Figure 2A-2B).

**Figure 3:**
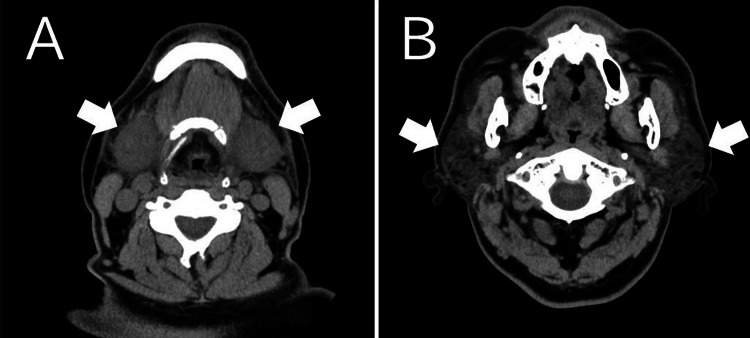
CT findings (HOD1). (A) Arrows indicate swelling of the bilateral submandibular glands (50 × 38 mm). (B) Arrows indicate swelling of the bilateral parotid glands (34 × 26 mm) CT: computed tomography, HOD1: hospital day 1

No sialoliths were detected. Additionally, a non-contrast neck CT scan confirmed swelling of the bilateral submandibular and parotid glands (Figure 3A-3B).

**Figure 4:**
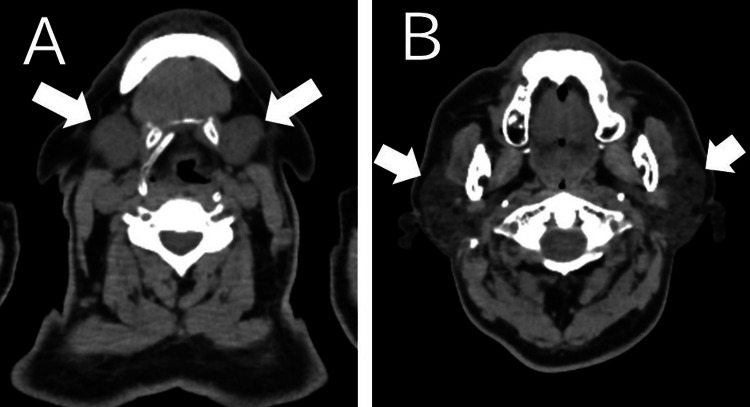
CT findings (HOD8). (A) Arrows indicate a reduction in bilateral submandibular gland size (44 × 25 mm). (B) Arrows indicate a reduction in bilateral parotid gland size (31 × 23 mm) CT: computed tomography, HOD8: hospital day 8

The patient was admitted for further evaluation of the underlying cause and monitoring for potential airway compromise. Additional blood test results did not suggest mumps virus infection (IgG positive, IgM negative). Autoimmune screening was negative for antinuclear antibodies, anti-SS-A, anti-SS-B, PR3-antineutrophil cytoplasmic antibodies (ANCAs), and MPO-ANCAs. His amylase level was elevated, suggesting a salivary gland origin. His total serum IgE level was also elevated (Table 1). Based on the delayed onset after contrast administration, absence of features suggestive of anaphylaxis, characteristic imaging findings, and exclusion of infectious and autoimmune etiologies, a diagnosis of CIS was made. The bilateral neck swelling gradually resolved without intervention. On HOD3, the swelling was no longer palpable, and on HOD5, his neck swelling had completely subsided. A follow-up non-contrast neck CT on HOD8 confirmed a reduction in submandibular and parotid gland swelling (Figure 4A-4B). Given his favorable clinical course, he was discharged on HOD9.

**Figure 5:**
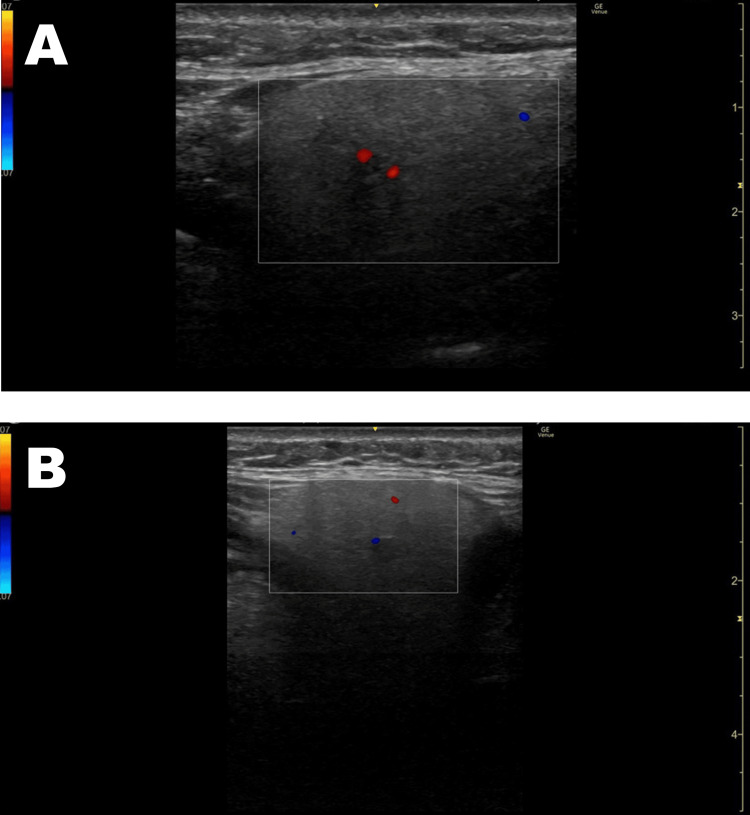
Neck color Doppler ultrasound findings (11 months after CIS recurrence). (A) Right submandibular gland. (B) Left submandibular gland Follow-up neck color Doppler ultrasound showed no salivary gland enlargement, tubular structures, or increased blood flow.

Four months later, he was scheduled for elective gallbladder polyp resection. As part of the preoperative evaluation, an outpatient contrast-enhanced abdominal CT scan was required. To prevent CIS recurrence, he received oral prednisolone (50 mg) at 13, 7, and 1 hour prior to contrast administration, along with a 500 mL saline infusion. He then underwent contrast-enhanced CT using 100 mL of iopromide 300. No immediate adverse effects were observed. However, at 10 hours after contrast administration, he again developed bilateral neck swelling and presented to the emergency department at our hospital. His vital signs remained stable, and there were no signs of anaphylaxis. The swelling gradually resolved spontaneously within two days. He subsequently underwent elective gallbladder polyp resection, recovered well postoperatively, and pathological examination of the resected gallbladder polyp revealed no malignant findings. Eleven months after the recurrence of CIS, we performed a follow-up neck ultrasound scan, which showed no salivary gland enlargement, no tubular structures, and no increased blood flow (Figure 5A-5B).

## Discussion

CIS is a relatively rare condition, and its pathophysiology and optimal management are not yet fully understood. In this case, the patient developed bilateral neck swelling after iodinated contrast administration, which resolved spontaneously. Despite corticosteroid premedication before re-exposure to contrast media for diagnostic purposes, salivary gland swelling recurred but again improved without intervention. This case is notable because CIS recurred despite corticosteroid premedication and the use of a different iodinated contrast agent.

CIS is characterized by transient swelling of the salivary glands following contrast media administration, most commonly affecting the submandibular and parotid glands. A meta-analysis by Jiao et al. reported a mean onset time of 16 hours, with the submandibular glands involved in 48% of cases and the parotid glands in 27% of cases [7]. In our case, swelling occurred 14 hours after contrast administration, involving the submandibular and parotid glands, consistent with typical CIS.

The exact pathogenesis remains unclear. The kidneys primarily excrete iodinated contrast media, and a small proportion may also be excreted through exocrine glands, including the salivary glands, where accumulation may lead to local inflammation and edema [5]. Jiao et al. described this reaction as a pseudo-allergic reaction [7]. Increased vascular permeability in the salivary glands, as suggested by the increased blood flow observed on neck ultrasound, likely reflects this acute inflammatory reaction [6]. This mechanism is distinct from that of IgE-mediated anaphylaxis, which occurs within minutes to hours and involves systemic symptoms. Although the patient’s serum IgE level was elevated, he had no history of atopic disease or known allergic disorders. Given the delayed onset of symptoms and absence of systemic manifestations of immediate hypersensitivity, this finding was considered incidental, and its relationship to CIS remains uncertain. As mild renal impairment was observed in this case, reduced renal clearance may have delayed elimination of the contrast agent and enhanced accumulation in the salivary glands, potentially contributing to the development of CIS.

Although the reported incidence of clinically apparent CIS is generally estimated at 1-2% [3], Lee et al. reported a higher incidence of 4.2% in a cohort of patients undergoing endovascular treatment for cerebrovascular disease [4]. These findings suggest that the true incidence of CIS may be underestimated because mild or subclinical cases may go unrecognized or unreported. In Japan, only two cases have been previously reported [1,2], which may reflect under-recognition of this condition domestically. Therefore, increased awareness of CIS among clinicians is important for accurate diagnosis and appropriate management.

A diagnosis of CIS requires exclusion of other conditions such as anaphylaxis, angioedema, infections, autoimmune diseases, and obstructive sialadenitis, including sialolithiasis. In our case, the patient developed symptoms 14 hours post-contrast administration, significantly later than the typical acute onset of anaphylaxis. Moreover, no skin or mucosal involvement, respiratory compromise, or hypotension was observed, and imaging confirmed salivary gland enlargement, making anaphylaxis and angioedema less likely. Laboratory test results were not consistent with mumps virus infection or autoimmune diseases, and ultrasound revealed no sialoliths, supporting the diagnosis of CIS.

Neck ultrasound is considered a valuable diagnostic tool in CIS. Lucarelli et al. described characteristic findings, including tubular structures with hypoechoic areas within the enlarged submandibular glands and increased central blood flow [6]. These features were also observed in our case and contributed to the diagnosis. At a follow-up neck ultrasound performed 11 months after the recurrence, these characteristic features had completely resolved, with no ductal structures or increased blood flow observed in either submandibular gland.

When using contrast media in patients with a history of CIS, the risks and benefits must be carefully considered. In this case, we used iopromide 300, a lower-iodine-concentration agent, and premedicated the patient with prednisolone using a regimen based on the American College of Radiology recommendations for contrast media premedication [8]. However, CIS recurrence occurred 10 hours post-contrast administration. Given that CIS is likely a pseudoallergic rather than IgE-mediated reaction, the effectiveness of corticosteroid premedication remains questionable. In addition, if CIS is primarily caused by contrast accumulation in the salivary glands and by local osmotic or toxic effects, corticosteroid premedication may not fully prevent its development.

The patient did not develop severe symptoms during the recurrence, and the symptoms resolved within two days, one day sooner than in the earlier episode. Therefore, corticosteroid premedication may have mitigated the severity or shortened the clinical course. However, because CIS often resolves spontaneously, this interpretation remains speculative. Dellapasqua et al. reported successful prevention of recurrence in a 60-year-old man who had previously developed CIS twice after contrast-enhanced CT by administering methylprednisolone 32 mg at 12 and 2 hours prior to contrast administration on the third occasion [9]. Therefore, the potential efficacy of corticosteroid premedication cannot be entirely dismissed. However, owing to the limited number of reported cases, further accumulation of clinical evidence is necessary to determine the role of prophylactic steroid use in CIS.

This case also highlights the benign and self-limited nature of CIS. The patient was hospitalized for nine days because of concern for potential airway compromise and the need to exclude other diagnoses. However, in clinically stable patients with typical features of CIS, no signs of airway compromise or anaphylaxis, and gradual symptom improvement, extensive diagnostic work-up or prolonged hospitalization may not be necessary. Increased awareness of CIS may help clinicians provide appropriate monitoring while avoiding unnecessary investigations and hospitalization.

## Conclusions

We report a rare case of recurrent CIS despite corticosteroid premedication and the use of a different iodinated contrast agent. CIS should be considered in patients who develop painless salivary gland swelling several hours after contrast media administration. In patients with a history of CIS, re-exposure to iodinated contrast media should be carefully considered, and patients should be informed of the possibility of recurrence. Although corticosteroid premedication may be considered, its preventive efficacy remains uncertain. Clinicians should be aware that CIS often follows a benign and self-limited course, but recurrence may occur despite preventive measures.
